# Investigation of Intestinal Absorption and Excretion of Paracetamol in Streptozotocin-Induced Hyperglycemia

**DOI:** 10.3390/ijms231911913

**Published:** 2022-10-07

**Authors:** Petra Mészáros, Sára Kovács, Győző Kulcsár, Melinda Páskuj, Attila Almási

**Affiliations:** 1 Institute of Pharmaceutical Chemistry, Faculty of Pharmacy, University of Pécs, Rókus utca 4, 7624 Pecs, Hungary; 2 Sandoz Novartis Division, 018953 Targu Mures, Romania

**Keywords:** paracetamol, acetaminophen, intestinal elimination, HPLC, streptozotocin, hyperglycemia, glutathione, cysteine

## Abstract

The phenolic drug molecules can be metabolized, among others, by the small intestine’s enterocytes. The conjugation reactions (glucuronidation and sulfation) show great importance in these transformations, although the oxidation reactions can be significant. These processes are dependent on the substituents of the phenolic compounds or the reacting functional groups (hydroxyl or carboxyl). Pathologic conditions, e.g., permanent hyperglycemia and diabetes, can alter the activities of the conjugative and possibly the oxidative enzymes, thus forming a change in the metabolic pattern and eventually provoking oxidative stress. A rat intestinal perfusion model was used to investigate the way in which experimental hyperglycemia affects the paracetamol’s intestinal elimination and metabolism. Hyperglycemia was induced by the administration of streptozotocin. Two hundred and fifty µM paracetamol was used in the intestinal perfusion solution. For the quantitation of the paracetamol and its major metabolites in the intestinal perfusate, an isocratic high-performance liquid chromatography method with UV-Vis detection was developed. The results revealed that quantities of all of the measured metabolites (glucuronide, sulfate, cysteine, and mercapturic acid conjugates) increased as the effect of the streptozotocin-induced hyperglycemia also did. In the small intestine’s homogenate, the glutathione levels showed that there was a decrease in the hyperglycemia levels after the paracetamol administration. In contrast, the tissue levels of the cysteine were lower in the streptozotocin-induced hyperglycemia and increased after the administration of the paracetamol. The changes in the activity of the intestinal CYP 3A4, CYP 2E1, and cyclooxygenase (COX) enzymes were determined in the control and the hyperglycemic cases. Still, there was a significant observable enzyme activity elevation in the intestinal COX enzymes, but there was a decrease in the amount of activity of the intestinal CYP3A4 enzymes, and the CYP2E1 enzyme activity was practically changeless. The results on the cysteine levels in the intestinal homogenate, at least partly, can be explained by the regulation function of the cysteine during the occurrence of oxidative stress.

## 1. Introduction

Phenolic derivatives are transformed mainly by conjugation (Phase II) reactions and by oxidation to a smaller (but sometimes functionally important) extent. While the conjugation processes mainly possess a detoxification effect, the derivatives of the oxidative transformations might interact with cellular macromolecules [1,2].

In earlier experiments, the phenolic 4-nitrophenol [3] and the arylpropionic acid ibuprofen were investigated. Metabolites that are excreted by the small intestinal enterocytes were only seen in the case of the administration of 4-nitrophenol. In the 4-nitrophenol administration experiment, only the conjugative metabolites were observable (glucuronide and sulfate), while the oxidative derivatives could not be detected [4]. In the ibuprofen administration experiment, the improper intestinal enzyme families and the relative instability of the ester-type glucuronide might be responsible for there being inappreciable quantities of the metabolites [5].

The paracetamol molecule contains an acetamido group that has an intermedier activating property since the resonance within the substituent competes with the delocalization of the lone pair into the ring. The influence of the phenolic hydroxyl group might alter the excess and the quality of the conjugation (and metabolic) process.

The intestinal tract is an important and less often investigated site of drug metabolism. Its location can be responsible for its significance because this is the site of the entry of the drugs, and the formed metabolites can be excreted both into the circulation and the gastrointestinal lumen [6,7].

The tested compound, paracetamol, in the USA, where it is also known as acetaminophen (*N*-acetyl-para-aminophenol, 4-hydroxy-acetanilide), is one of the most widely used antipyretic and analgesic drugs among many of the over-the-counter medicines. Paracetamol is unique among analgesic drugs because it has a very weak anti-inflammatory effect and it is free from some of the typical side effects such as a reduced defense of the gastrointestinal mucosa and cardiorenal effects [8,9]. The mechanism of action of it is also specific in comparison to those of other non-steroidal anti-inflammatory drugs (NSAIDs). Additionally, after a decade, the mechanism of action of it is still under investigation [10,11,12,13].

Paracetamol is a moderately water soluble (partition coefficient between octanol and water is 3.2) weak acid (pKa = 9.5–9.7), therefore, it is mostly unionized at a physiological pH. It has a low molecular mass, so to sum up, the absorption of paracetamol mostly occurs by passive diffusion [14,15]. Paracetamol is absorbed in the proximal part of the small intestine, mostly in the duodenum and jejunum [15], but theoretically it can be absorbed in the entire length of the gastrointestinal tract. It has a high oral bioavailability, and within 90 min, it reaches its peak concentration. In the case of rats, 70% of the paracetamol was absorbed in the small intestine in 30 min [16,17].

The metabolism process of paracetamol is well known; after therapeutic dose, the paracetamol is converted—mostly in the liver—into inactive paracetamol β-D-glucuronide, paracetamol sulphate, and a minor fraction is formed into a reactive metabolite, *N*-acetyl-p-benzoquinone imine (NAPQI), and less than 5% of it is excreted in the urine and this is unchanged [17,18]. This highly reactive metabolite is mainly produced by a CYP3A4, CYP2E1, CYP1A2, and cyclooxygenase (COX)-mediated enzymatic transformation and it is inactivated by a conjugation with glutathione; the conjugate ultimately transforms into paracetamol cysteine and then, it transforms into paracetamol mercapturate conjugates [19,20,21]. The cellular presence of cysteine can also play an important role in the elimination of the formed reactive species and give information on the detoxifying ability of the cell; cysteine insufficiency leads to a collapse of the intracellular glutathione level [22].

Prolonged hyperglycemia can provoke the formation of reactive oxygen species (ROS), and consequently, the production of the oxidizing derivatives are elevated, and the efficiency of the antioxidant defense is reduced [23,24]. The levels of the free radicals and reactive species are reduced by the glutathione directly or enzymatically [25]. The presence of the reactive oxidative agents can be lowered by the cysteine that is present in the cells in the bonded or non-bonded forms [26]. The onset of the hyperglycemia also influences the expressions and activity of P-gp, MRP-2, BCRP and the activity of various metabolic enzymes (uridine glucuronyltransferase and sulfotransferase) in the metabolizing organs [4].

The present investigation was designed to study the effect of hyperglycemia on the intestinal metabolism of paracetamol, the activities of the involved enzymes, and on the luminal appearance of its major metabolites. An investigation of the function of the thiol defense system was also conducted in rats in control groups, in hyperglycemic groups, and after a paracetamol administration in both of these groups. These results can add more pieces of information to the interpretability of the metabolic changes of the phenolic compounds with substrates possess different electron attractive forces.

## 2. Results

### 2.1. Validation of a Chromatographic Method to Determine the Metabolites in Perfusate

#### 2.1.1. Specificity

**Figure 1:**
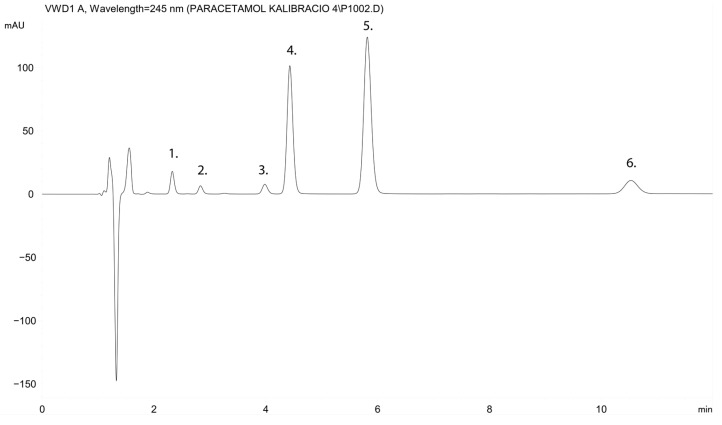
HPLC chromatogram of the standards prepared with the blank perfusate. Peak 1 is paracetamol β-D-glucuronide (t_R_ = 2.305 min), peak 2 is paracetamol cysteine (t_R_ = 2.776 min), peak 3 is paracetamol sulphate (t_R_ = 3.904 min), peak 4 is paracetamol (t_R_ = 4.354 min), peak 5 is theophylline (internal standard) (t_R_ = 5.701 min), and peak 6 is paracetamol mercapturate (t_R_ = 10.271 min).

The specificity was defined as the ability of the methods to differentiate and quatify the analytes in the presence of the endogenous constituents of the perfusate and the samples. Figure 1 shows the standards that were prepared with the blank perfusate. There were no endogenous peaks in the regions of the retention times of the paracetamol, paracetamol β-D-glucuronide, paracetamol sulphate, paracetamol cysteine nor the paracetamol mercapturate and the internal standard theophylline (Figure 1).

#### 2.1.2. Linearity

The linearity of the method was studied by preparing a series of paracetamol in the range of 10–1000µM (10, 50, 100, 500, and 1000 µM), paracetamol β-D-glucuronide in the range of 2–100 µM (2, 5, 10, 50, and 100 µM), paracetamol sulphate in the range of 2–10 µM (2, 4, 6, 8, and 10 µM), paracetamol cysteine in the range of 2–10 µM (2, 4, 6, 8, and 10 µM), and paracetamol-mercapturate in the range of 2–10 µM (2, 4, 6, 8, and 10 µM). Data were obtained from five parallel injections at each concentration level. The calibration curves were made by plotting the theoretical concentrations against the relative peak areas. The linearity was determined by least-squares regression method. The regression equation for the paracetamol was y = 0.0064x − 0.0032 (R^2^ = 1), for the paracetamol β-D-glucuronide, it was y = 0.0073x + 0.0032 (R^2^ = 0.9995), for the paracetamol sulphate, it was y = 0.0076x + 3 × 10^−5^ (R^2^ = 0.9999), for the paracetamol cysteine, it was y = 0.0054x − 0.0009 (R^2^ = 0.9985), and for the paracetamol mercapturate, it was y = 0.0272x − 0.008 (R^2^ = 0.9994).

#### 2.1.3. Precision

The precision of the high-pressure liquid chromatographic method was examined by the determining the intraday precision and the interday precision of the results.

The intraday precision of the retention times and the integrated peak areas were determined by measuring five concentrations of paracetamol and its metabolites in a drug-free intestinal perfusate. Each concentration was analyzed five times.

To determine the interday precision of the retention times and integrated peak areas, five different concentrations of paracetamol and its metabolites were prepared and measured in a drug-free intestinal perfusate for working days over five consecutive weeks.

The percent RSDs of the interday precision of the retention times of paracetamol were RSD = 1.06–1.65; while those of the integrated peak areas were RSD = 1.63–7.30. The corresponding values were RSD = 0.81–1.21 and RSD = 1.20–8.06 for the paracetamol β-D-glucuronid, RSD = 1.29–2.13 and RSD = 2.31–8.35 for the paracetamol sulphate, RSD = 1.27–1.92 and RSD = 1.69–8.12 for the paracetamol cysteine, and RSD = 1.73–2.72 and 2.81–8.30 for the paracetamol mercapturate, respectively (Table A1, Table A2, Table A3, Table A4 and Table A5).

The precision of the peak areas were calculated from the ratios of the peak areas of the paracetamol mercapturate and theophylline. The values represent the mean of five independent measurments.

#### 2.1.4. Determination of the Limits of Detection and Quantification

Based on the calculation of the root mean square error (RMSE) for the concentration of the paracetamol in a range of 10–100 µM, the limit of detection was found to be 5.84 µM in the small intestinal perfusate (calculated as 3 × RMSE/m, where m is the slope of the calibration curve). Based on the calculation of the RMSE for the concentration (2–10 µM range) of paracetamol β-D-glucuronide, the limit of detection was found to be 0.61 µM. The limit of detection for paracetamol sulphate was 0.29 µM, while for paracetamol cysteine, it was 0.35 µM, and for paracetamol mercapturate, it was 0.13 µM.

Based on the calculation of the root mean square error (RMSE) for the concentration of the paracetamol in a range of 10–100 µM, the limit of quantification was found to be 48.83 µM in the small intestinal perfusate (calculated as 10 × RMSE/m, where m is the slope of the calibration curve). Based on the calculation of the RMSE for the concentration (2–10 µM range) of paracetamol β-D-glucuronide, the limit of quantification was found to be 2.02 µM. The limit of detection for paracetamol sulphate was 0.96 µM, while for paracetamol-cysteine, it was 1.17 µM, and for paracetamol mercapturate, it was 0.42 µM.

#### 2.1.5. Identification of the Metabolites by HRMS Measurement

The developed HPLC method was suitable for monitoring the product metabolism of the paracetamol, while the products in the incubate were identified based on their chromatographic retention times, and their structure was verified by the mass spectrometric measurements (Figure A3, Figure A4, Figure A5, Figure A6 and Figure A7). To identify the structure of the formed metabolites in the incubates, a Dionex Ultimate 3000 HPLC system (Dionex, Sunnyvale, CA, USA)—which was used according to the conditions that are detailed in the HPLC and MS measurements section—connected to a mass spectrometer was used. Mass spectrometry was performed using a Thermo High Resolution Q Exactive mass spectrometer (Thermo Fisher Scientific, Bremen, Germany) and a Heated Electrospray Ionization Source II (HESI II). The identified metabolites were as follows: paracetamol β-D-glucuronide (323.101 Da) (Figure A3), paracetamol sulphate (232.026 Da) (Figure A4), paracetamol cysteine (271.073 Da) (Figure A5), paracetamol glutathione (457.136 Da) (Figure A7), and paracetamol mercapturate (313.083 Da) (Figure A6), respectively.

#### 2.1.6. Calculations and Statistical Analysis

Data are presented as the means ± SE of five independent experiments. The difference among the groups was determined using a Student’s *t*-test. The experimental values were considered to be significantly different from the control value at *p* < 0.05.

### 2.2. Figures

Streptozotocin (STZ) has a diabetogenic property in the islet β-cell destruction. Many different STZ-dosing regimens are used to produce a model of type 1 and type 2 diabetes mellitus [27,28]. In this experiment, a single dose of 65 mg/kg (i.v.) was used. The hyperglycemia was confirmed after 1 week of the rats receiving a single STZ treatment. The mean blood glucose level in the control rats was 7.34 ± 0.114 mM, while that of the STZ-pretreated animals was 23.78 ± 2.593 mM.

**Figure 2:**
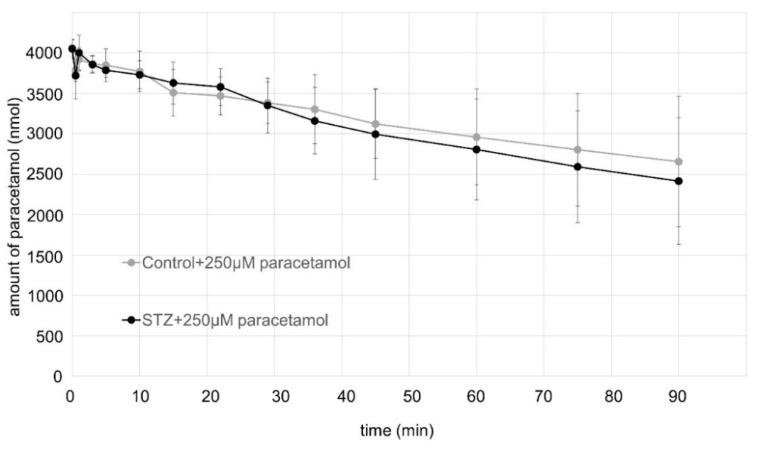
Time course of the disappearance of paracetamol in rat small intestinal perfusate during perfusion of the jejunal loop with isotonic medium containing 250 µM paracetamol. Each value represents the mean of 5 independent experiments ± standard error.

Figure 2 shows the time course of the disappearance of the paracetamol from the perfused rat jejunal segment of the small intestine of the control and the STZ-pretreated rats. The validated HPLC UV-Vis method detected a slow decrease in the disappearence of the paracetamol in the small intestinal perfusates during the experiments. There were no significant difference between the control and the hyperglycemic rats.

**Figure 3:**
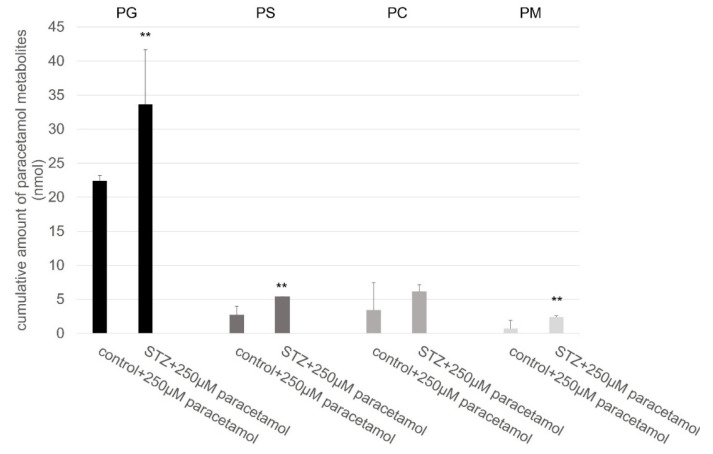
Changes in the excreted amount of paracetamol β-D-glucuronide (PG), paracetamol sulphate (PS), paracetamol cysteine (PC), and paracetamol mercapturate (PM) into the lumen of the small intestine of rat during perfusion of the jejunal loop with isotonic medium containing 250 µM paracetamol. Each value represents the mean of 5 independent experiments ± standard error. **, *p* < 0.01 versus control and STZ pretreated groups.

Figure 3 shows the cumulative excreted amount (sum of excreted amounts in 90 min) of the paracetamol β-D-glucuronide (PG), paracetamol sulphate (PS), paracetamol cysteine (PC), and paracetamol mercapturate (PM). The STZ pretreatment significantly increased the excretion of the paracetamol β-D-glucuronide, paracetamol sulphate, and paracetamol mercapturate into the lumen of the small intestine. There were no significant differences between the control and the hyperglycemic rats in the case of paracetamol-cysteine. The growth of the observed metabolites was consistent in the 90 min duration of the experiment.

**Figure 4:**
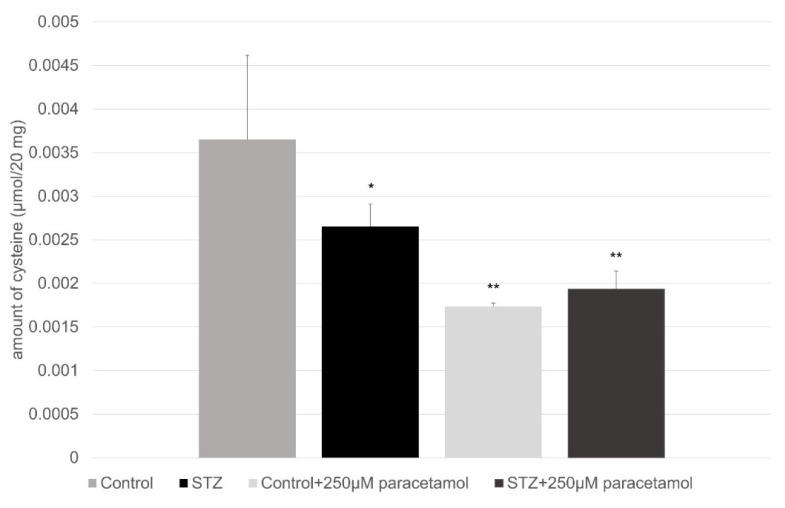
Amount of cysteine (µmol/20 mg) in the small intestine without and with paracetamol perfusion in case of control and diabetic rats. Values represent the mean ± S.E. of five rats. Each value represents the mean of 5 independent experiments ± standard error. *, *p* < 0.05; **, *p* < 0.01 versus control.

Figure 4 shows the amount of cysteine (µmol/20 mg) that was in the small intestinal perfusate in the four groups of treatments: without and a paracetamol perfusion in the case of control and STZ-pretereated rats. The streptozotocin pretreatment significantly reduced the level of the cystein, while after the paracetamol perfusion in the corresponding line-up, a parallel change was not detectable.

**Figure 5:**
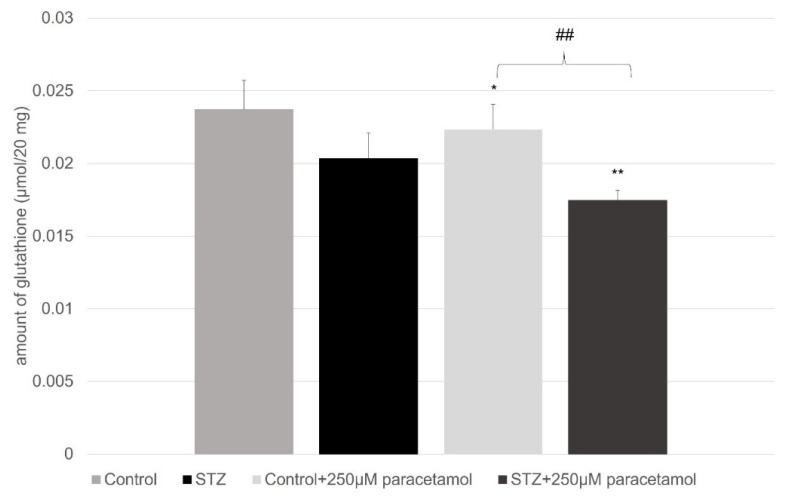
Amount of glutathione (µmol/20 mg) in the small intestine without and with paracetamol perfusion in case of control and diabetic rats. Values represent the mean ± S.E. of five rats. Each value represents the mean of 5 independent experiments ± standard error. *, *p* < 0.05; **, *p* < 0.01, ^##^, *p* < 0.01 versus control.

Figure 5 represents the concentrations of the glutathione (µmol/20 mg) that were in the small intestinal perfusate in the four groups of treatments: without and with the paracetamol perfusion in the control and STZ-pretreated rats. The STZ pretreatment significantly reduced the level of the glutathion, while after paracetamol administration, a further decrease was observable.

**Figure 6:**
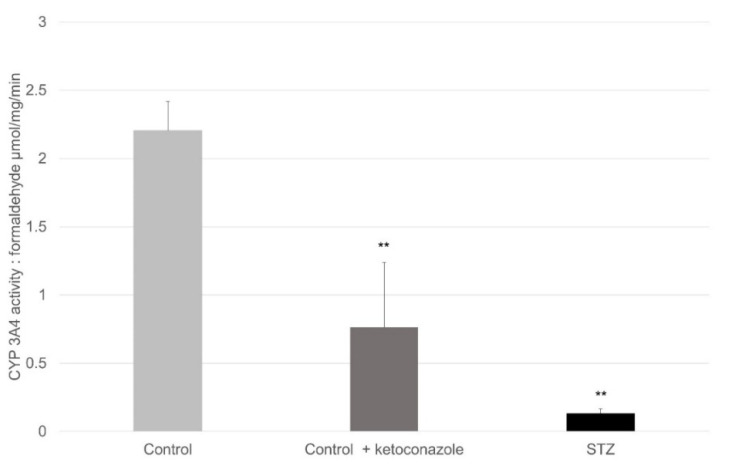
Activity of CYP 3A4 of the small intestine in control, positive control and diabetic rats. Values represent the mean ± S.E. of five rats. Each value represents the mean of 5 independent experiments ± standard error. **, *p* < 0.01 versus control. STZ, steptozotocin. The enzyme activity is expressed in μmol formaldehyde/mg/min, which was conjugated in 1 min which was calculated for 1 mg protein of the small intestine.

Figure 6 shows the activities of CYP 3A4 in the proximal segment of the small intestine. The amount of activity of CYP 3A4 was significantly decreased in the diabetic rats. Ketoconazole is an enzyme inhibitor, and it was applied to the results to create a positive control. These samples originated from animals that were not involved in the paracetamol perfusion experiment.

**Figure 7:**
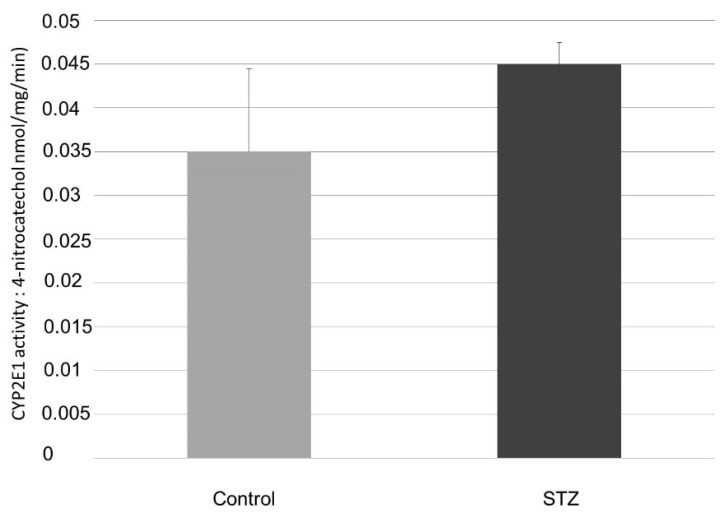
Activity of CYP 2E1 of the small intestine in control and diabetic rats. Values represent the mean ± S.E. of five rats. Each value represents the mean of 5 independent experiments ± standard error. The enzyme activity is expressed in nmol 4-nitrocatechol/mg/min.

Figure 7 shows the activities of the CYP 2E1 enzyme in the proximal segment of the small intestine. The amount of activity of CYP 2E1 was not changed significantly by the streptozotocin pretreatment. This observation was also seen in the samples of the control and STZ-pretreated animals.

**Figure 8:**
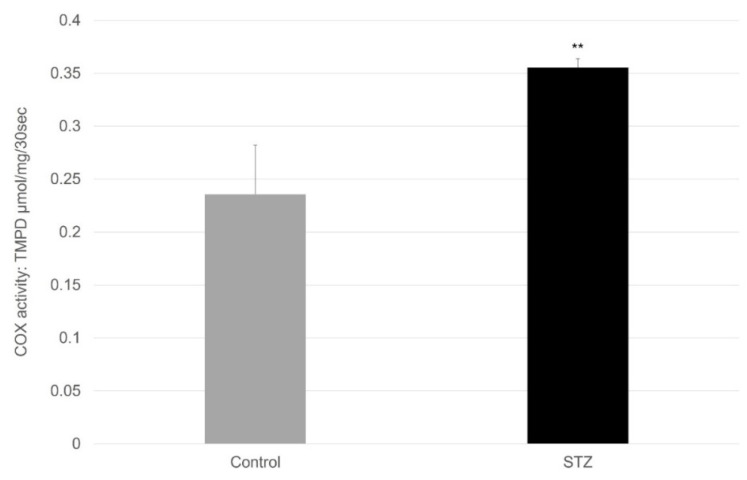
Activity of COX of the small intestine in control and diabetic rats. Values represent the mean ± S.E. of five rats. Each value represents the mean of 5 independent experiments ± standard error. **, *p* < 0.01 versus control. STZ, steptozotocin. The enzyme activity is expressed in μmol TMPD/mg/min, which was conjugated in 30 s calculated for 1 mg protein of the small intestine.

Figure 8 shows the activities of the COX enzyme in the small intestine. The amount of activity of COX was significantly increased in the diabetic rats.

## 3. Discussion

**Figure A2:**
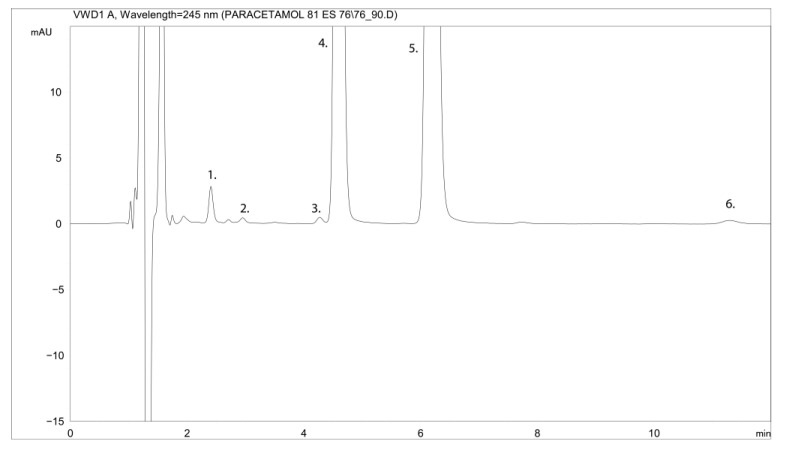
HPLC chromatograms the perfusate generated in rat small intestine luminal after 90 min of perfusion. Peak 1 is paracetamol β-D-glucuronide, peak 2 is paracetamol cysteine, peak 3 is paracetamol sulphate, peak 4 is paracetamol, peak 5 is theophylline (internal standard), and peak 6 is paracetamol mercapturate.

The isocratic, validated RP HPLC UV-Vis method was developed for the quantification of paracetamol and its major metabolites (glucuronide, sulphate, cysteine, and mercapturate) from small intestinal perfusate samples to provide rapid measurements with a retention time < 12 min (Figure 1 and Figure A2). Other authors have described methods to quantify the two major metabolites (glucuronide and sulphate) in urine and plasma [29,30,31], while some publications have introduced methods to quantify the four major metabolites (glucuronide, sulphate, cysteine, and mercapturate) in plasma [32] or some have used methods with a higher retention time in both plasma and urine [33] such as the ion-pair gradient method in plasma [34].

Figure 2 shows that the administered amount of paracetamol slowly but continuously decreased during the 90-min-long experiment. The pKa value (9.4) of the paracetamol suggests that the paracetamol molecules were in a neutral form for the full length of the gastrointestinal tract and could be absorbed by a passive diffusion. The area that was investigated was relatively short (10 cm), i.e., the jejunal segment—the length that was investigated was originally determined by the experimental setup, wherein, the disappearance of the parent compound was rather slow—however, this process can be applied to further segments and regions of the intestinal tract [15]. The absorption curve represents that the disappearance of the paracetamol is slightly faster in the streptozotocin-pretreated animals, although this change is not significant, nonetheless, this tendency seems to be a consequence of the experiment. The hyperglycemia can form an intestinal barrier disfunction, thus causing an elevated permeability [35], although the paracetamol itself also can positively influence the membrane integrity [36], whereby the final result is to be dependent from the balance of the two factors.

The small intestine is an organ with a lower rate of metabolic activity in comparison with the liver, although it is more accessible for in vivo experiments than other organs are, and this makes it possible to investigate several fine changes in the metabolic processes. If other regions are considered, then it must be noted that these various isolated metabolizing organs have evolved different metabolic profiles, i.e., in the liver, in the case of an intravenous administration, around the one third of the administered paracetamol dose is excreted into the bile; approximately 50% is excreted as glucuronide, 30% is excreted as sulfate, 15% is excreted as glutathione, and 1% is excreted as mercapturate, while the rest of it remains as a parent compound [37,38], whereas the kidney has a weak metabolic capacity as an isolated organ; only 10% of the administered paracetamol is metabolized, and sulfate, glucuronide, cysteine, and mercapturate conjugates appear [17,39].

**Figure A3:**
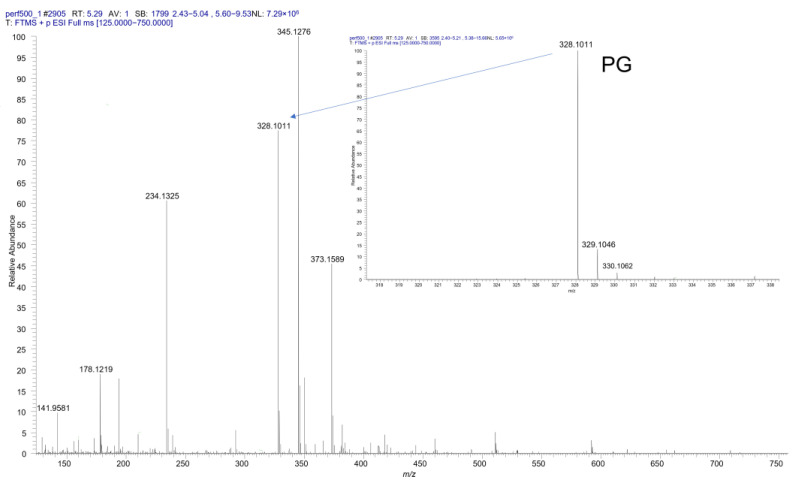
HRMS spectrum of paracetamol β-D-glucuronide generated in rat small intestine luminal after 90 min of perfusion.

**Figure A4:**
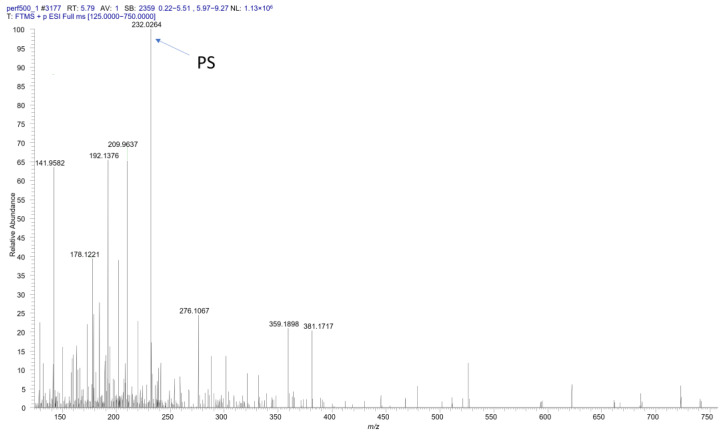
HRMS spectrum of paracetamol sulphate generated in rat small intestine luminal after 90 min of perfusion.

**Figure A5:**
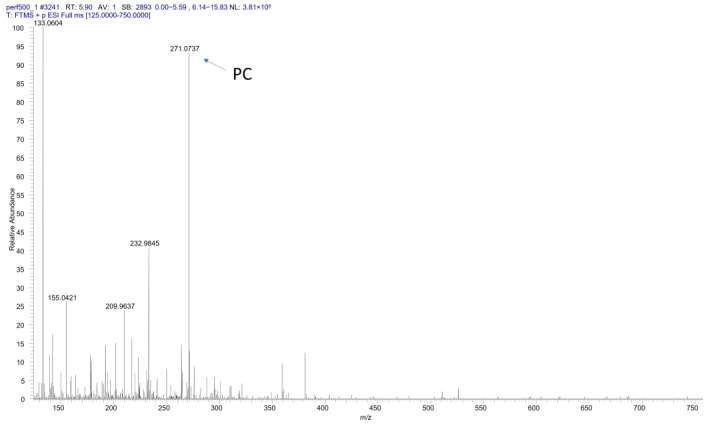
HRMS spectrum of paracetamol cysteine generated in rat small intestine luminal after 90 min of perfusion.

**Figure A6:**
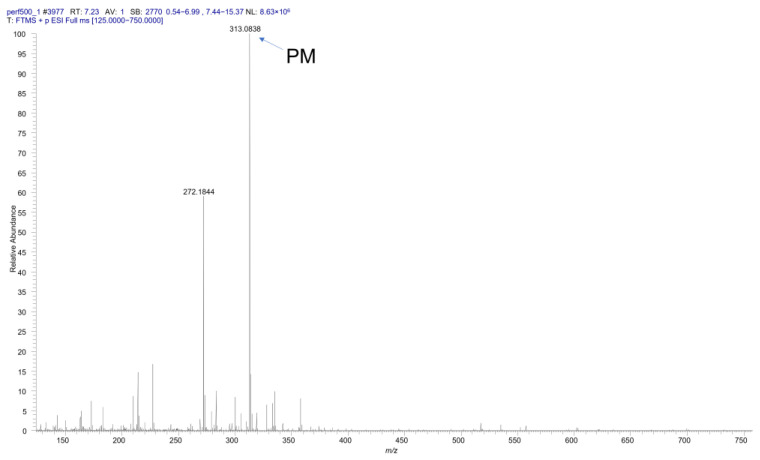
HRMS spectrum of paracetamol mercapturate generated in rat small intestine luminal after 90 min of perfusion.

**Figure A7:**
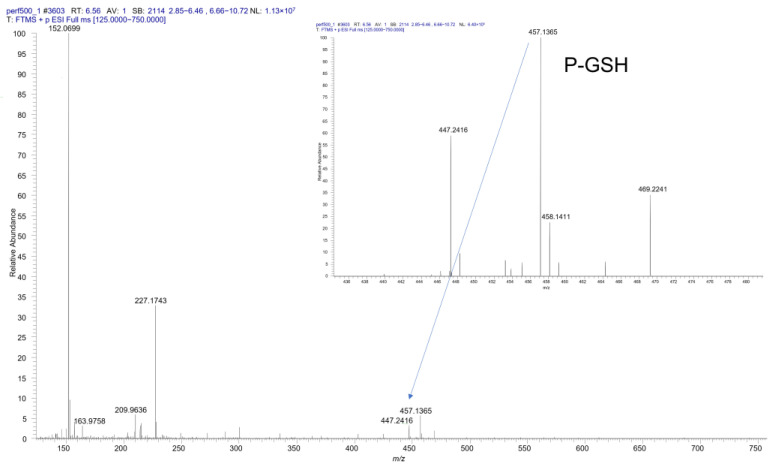
HRMS spectrum of paracetamol-glutathione generated in rat small intestine luminal after 90 min of perfusion.

In the intestinal perfusate, several metabolites are excreted aside from the glucuronide and sulfate conjugates (main derivatives) by the enterocytes of the intestinal wall, and these metabolites are originated from oxidation processes. While the appearance of the glutathione conjugate can be attributed to the glutathione pool and the detoxifying function of the glutathione, the presence of the cysteine conjugate (PC) is derived from the glutathione degradation and from the actual cysteine level, and the continuous cysteine formation is derived from the extracellular cystine pool [40]. The presence of the metabolites (PG, PS, PC, PM, and PGSH) were confirmed by the LC-MS method (Figure A3, Figure A4, Figure A5, Figure A6 and Figure A7).

The glucuronide conjugate makes up the majority of the formed metabolites, and it is a relatively stable ether-type conjugate. The dominance of the glucuronide conjugate corresponds with the metabolic pattern of 4-nitrophenol, forming an ether-type conjugate with UGT1A1, UGT1A6, and UGT1A8 enzymes. The formation of the sulfate conjugate is more restrained, but it appears in a higher amount in comparison with the metabolic sulfate derivative of the 4-nitrophenol. In 4-nitrophenol, the ring of the phenol is substituted with a more electron-withdrawing nitrate group [1]. Both of the glucuronide and sulfate conjugates show that they have an elevated level in the experimental diabetes group. These elevations at least partly can be explained by the increase of the activities of the UDP-glucuronyltransferase and sulfotransferase enzymes in the small intestinal wall [40].

Only very few data exist on the small intestinal enzyme activities in STZ-induced diabetes or in connection with the intestinal metabolism of phenolic compounds with a moderately activating acetamido group. The appearance of the metabolites can be the result of several factors [41], and the detected results can show that there is variability between the organs. The rate of the conjugative enzyme activities (glucuronyltransferase and sulfortransferase) can show an increase in the small intestinal wall one week after the STZ administration, which is the opposite of the activity rate decrease that occurs in the liver. The activity changes of the oxidative enzymes show a varied picture that is based on the corresponding references. The amount of CYP2E1 enzyme activity has been shown to increase in rat livers [42] and increase in human lymphocytes [43], while it has been changeless [44] or it has decreased in the kidneys of mice [42].

The activity of the CYP3A4 enzymes show an increase in rat livers [45], although a decrease was observed in human samples [41]. In mice kidneys, the amount of activity for CYP3A4 shows an elevation by the effect of the STZ pretreatment [42]. Regarding the COX enzyme activities, hyperglycemia and COX-2 activation can be a causative factor of diabetic complications [46].

The paracetamol can be oxidized by the CYP3A4, CYP2E1, and CYP1A2 enzymes in the liver [47]. In the small intestine, almost exclusively, the CYP3A4 [48] is expressed in a higher proportion, so it seems to be most important contributor to the formation of the reactive paracetamol metabolite, the *N*-acetyl-p-quinone-imine (NAPQI). Some publications have emphasized the minor presence of the CYP2E1 enzyme also [49,50]. NAPQI also can be formed by the intestinal cyclooxygenase enzymes [21].

Based on the findings for the small intestine in Figure 6, Figure 7 and Figure 8, the elevated levels of the oxidative products can only be explained by the activity changes of the cyclooxygenase enzymes. In the case of inflammations and in cases with elevated peroxide radical formation (in diabetes and after STZ pretreatment), the COX-2 activity can be increased, and the paracetamol has only a little effect on it [8]. The amount of activity of the CYP3A4 enzyme decreased, while the amount of activity of the CYP2E1 enzyme was extremely low and practically changeless.

The present results show that the levels of the paracetamol cysteine (PG) and paracetamol mercapturate (PM) metabolites in the small intestinal perfusates had risen after a one-week STZ pretreatment.

The hyperglycemia and the experimentally provoked diabetes induced an elevated oxidative stress, which was followed by there being high levels of free radicals and the simultaneous decline of the antioxidant defense mechanisms [51]. The non-enzymatic oxidative processes can produce hydroquinone, p-benzoquinone, p-aminophenol, p-nitrophenol, and benzaldehydes from paracetamol, and these derivatives interact with the cellular glutathione and cysteine [52]. When it is viewed as a whole, the overall status of the thiol redox system can reflect important information on the condition and reserve pool of the antioxidant defense mechanisms. The thiol defense mechanism can be made up of several thiol-containing structures, and several parallel processes can maintain its constituting factors. The changes in the levels of these thiol-containing structures can be contrary to the predictable molecular progress of the oxidative stresses that are causing the initial decline of these substrates. In Figure 4 and Figure 5, the influence of hyperglycemia and the paracetamol (and the formed reactive agents) on the actual levels of the free glutathione and cysteine can be seen. It is well visible that the STZ-provoked oxidative stress and the paracetamol administration alone lowered the levels of the glutathione and cysteine, however when the paracetamol was administered to the STZ-pretreated rats, a slight elevation of the cysteine level occurred. Altogether, because of the rapid mobilization of the cysteine, the oxidative stress can upregulate the capacity of the thiol redox defense system [53].

In the intestinal perfusate samples, the PC conjugate and the PM can be detected with the applied HPLC-UV method. These products can be considered to be the result of the reaction of the NAPQI and glutathione, cysteine, and the decomposition and acetylation of them. The free cysteine can be obtained as an intermedier of the glutathione formation [54,55], and it can be derived from methionine, and a continuous cysteine flux also exists based on the cystein/cystin redox cycle. The food-derived cystine also can make up the reserves for the cysteine supplementation. The cysteine itself has an important role in the maintenance of the physiological redox potential inside/outside of the cell. The oxidative stress induces the cystine/glutamate transport system and cystine enters the cell. This system has a GSH-independent redox system by sustaining a redox cycle over the plasma membrane. The function of this cycle is the cystine uptake, a reduction of the cystine to cysteine, and the removal of the surplus cysteine. The most active cystine transporter in the cell membrane is expressed on the brush border of the kidneys and the small intestine [56]. The NAPQI metabolite can cause the irreversible inhibition of the glutathione synthetase enzyme in vitro, this process also can contribute to the elevation of the cysteine level in the tissue homogenate after the administration of the paracetamol [57].

The decomposition of the glutathione conjugate rapidly and effectively occurs in the kidneys, liver, and in the intestinal tract. Specifically in rats, the hepatic γ-glutamyltransferase has a lower rate of activity, and this way, the liver plays a diminished role in the degradation of the glutathione conjugate to mercapturate. The small intestine can also take part in the transformation of the glutathione conjugates that are excreted into the bile [58,59].

After the degradation of the glutathione, the paracetamol cysteine conjugate is slowly acetylating as it is shown by the isolated intestinal cells [59], although some references emphasize the inter-organ feature of the process also [60]. In the gastrointestinal tract of a rat, a relatively homogenous longitudinal distribution of the *N*-acetyltansferase was observed [61]. The level of the paracetamol cysteine conjugate in the biological fluids can be an indicator of the intoxication because it can be released following cell necrosis [62].

## 4. Materials and Methods

### 4.1. Chemicals and Reagents

Paracetamol, urethan, sodium citrate dehydrate, citric acid, triethylamine, nicotinamide adenine dinucleotide phosphate (NADPH), glutathione (GSH), Tris hydrochloride, *N*,*N*,*N*′,*N*′-tetramethyl-p-phenylenediamine (TMPD), arachidonic acid (AA), and streptozotocin (STZ) were purchased from Sigma–Aldrich (Budapest, Hungary); potassium chloride (KCl), calcium chloride (CaCl_2_·2H_2_O), sodium chloride (NaCl), glucose, sucrose, EDTA disodium salt, ammonium acetate, glacial acetic acid, acetylacetone, potassium hydroxide (KOH), glycerol, ethanol, formaldehyde, and disodium hydrogen phosphate (Na_2_HPO_4_·2H_2_O) were purchased from Molar Chemicals Kft. (Halásztelek, Hungary); magnesium sulfate (MgSO_4_), theophylline, and acetonitrile were purchased from VWR International (Radnor, PA, USA); potassium dihydrogen phosphate (KH_2_PO_4_), dipotassium hydrogen phosphate (K_2_HPO_4_), L-cysteine hydrochloride, 5,5′-dithio-bis-[2-nitrobenzoic acid] (DTNB), metaphosphoric acid (HPO_3_), and mannitol were purchased from Reanal Labor (Budapest, Hungary); 4-acetamidophenyl β-D-glucuronide sodium salt, 4-acetaminoiphen sulfate potassium salt, 3-cyteinylacetaminophen trifluoroacetic acid salt, 3-(*N*-acetyl-L-cysteine-S-yl)-acetaminophen, erythromycin estolate, and ketoconazole were purchased from Toronto Research Chemicals (Toronto, ON, Canada). The solvents were of HPLC grade. The standard isotonic perfusion medium had the following composition: 96.4 mM NaCl, 7.0 mM KCl, 3.0 mM CaCl_2_, 1.0 mM MgSO_4_, 0.9 mM sodium phosphate buffer (pH 7.4), 29.5 mM Tris buffer (pH 7.4), 14.0 mM glucose, and 14.0 mM mannitol. Distilled water was purified using a Purelab Option Q7 Water System (ELGA LabWater, Woodridge, IL, USA) at the Department of Pharmaceutical Chemistry, University of Pécs. A WTW InoLab pH 7110 pH meter and a WTW SenTix 41 (Xylem Analytics, Weilheim, Germany) electrode were used to adjust the pH. Blood glucose level was controlled using an AccuChek blood glucose meter (Roche, Basel, Switzerland).

### 4.2. Animals and Experimental Procedure

Male Wistar-Hanover rats (weighing 240–300 g) were used in this experiment. The rats were anesthetized with urethane (1.2 g/kg, i.p.). The abdomen was opened by a mid-line incision, and a jejunal loop (length about 9–12 cm) was isolated and cannulated. The lumen of the jejunal loop was flushed with a warmed isotonic solution (30–40 mL) to remove digesta and food residues and then, blown empty with 4–5 mL air. Perfusion through the lumen of the jejunal loop with a 250 µM solution of paracetamol was performed at the rate of 12 mL/min in a recirculation mode for 90 min. The initial perfusion volume was 16.5 mL. During the 90-min-long experiment, 250 µL samples were collected. The temperature of perfusion was maintained constant at 37 °C. The samples were stored in refrigerator (−70 °C) until the analysis was performed and were measured in two weeks.

Experimental diabetes was induced by intravenous administration of streptozotocin at a dose of 65 mg/kg (dissolved in 0.1 M citrate buffer, pH 4.0) 1 week before the experiment. Control rats were treated with a vehicle. Blood glucose was measured before the STZ treatment and after 1 week before the start of the experiments. The experimental animals were provided with standard rat chow (ToxiCoop, Budapest, Hungary). Food was withdrawn 12 h before glucose levels were tested. Small intestine perfusate and bile samples were obtained from the same rats in 2 different groups (control and pretreated with STZ). The half of the animal groups got only blank perfusate, while for the other half of the groups, 250 µM paracetamol was perfused. Each group had 5 rats. After the experiments, the animals were over-anesthetized by urethane.

### 4.3. HPLC and MS Measurements to Determine the Metabolites in Perfusate

Before the analysis was performed, the collected perfusate samples (250 µL) were allowed to reach the ambient temperature and then vortexed for 5 s. After vortexing, 75 µL of each sample was mixed with 25 µL of 0.00159 M theophylline which was dissolved in Krebs-Tris buffer as an internal standard. The samples were vortexed for 5 s again, and then 10 µM of 3.88 M HClO_4_ solution was added. The samples were vortex-mixed for 5 s repeatedly and then centrifuged at 10,000× *g* for 10 min to sediment the precipitated protein. After centrifugation, 50 µL was transferred and mixed with 5 µL of 3.96 M NaOH. After the vortexing and centrifugation processes, a 20 µL sample was injected.

Analysis of the perfusates was performed using Agilent 1100 HPLC system (Agilent Technologies, Santa Clara, CA, USA) which was equipped with a quaternary HPLC pump (G1311A), degasser (G1322A), an autosampler (G1313A), a thermostated column compartment (G1316A), and a UV-Vis detector (G1314A). Data were recorded and evaluated using Agilent ChemStation software (Rev.B.03.02-SR2). Reversed-phase PerfectSIL 120 ODS C18 (4.6 mm × 100 mm, 5 µm particle size) (MZ-Analysentechnik GmbH, Mainz, Germany) were used for analytical separation.

The mobile phase consisted of water: acetonitrile: triethylamine (92.95:7.00:0.05 *v*/*v*%) with pH of 2.25 that was adjusted with formic acid. The flow rate of the eluent was 1 mL/min. The volume of the samples was 20 µL, and the measurements were done at ambient temperature. Detection processes were performed at a wavelength of 245 nm. Standard solutions were prepared by adding a known concentration of substance that was prepared in phosphate buffer and added to the drug-free perfusate. The standard solutions contained 0.00159 M theophylline as the internal standard. The constructed calibration curves were based on the ratio of the integrated peak areas of the paracetamol standards and the internal standard. Five replicate injections of the standard solutions were made. The relative standard deviation (RSD) of peak areas and retention times was calculated to assess the intraday precision. The same solutions stored at −70 °C were used to determine inter-day assay variations.

To identify the structure of the metabolites, a Thermo Dionex UltiMate 3000 liquid chromatograph connected to a Thermo Q Exactive Focus quadrupole-Orbitrap hybrid mass spectrometer was used. Data acquisition was carried out using Q Exactive Focus 2.1 and Xcalibur 4.2 software (Thermo Fisher Scientific, Waltham, MA, USA).

The HPLC separation was performed on an Accucore-C18 analytical column (150 mm × 2.1 mm, 2.6 µm) using Accucore-C18 precolumn (5 mm × 2.1 mm, 2.6 µm) at 40 °C. The injection volume was set to 5 µL, the flow rate was set to 0.3 mL/min, and the autosampler vials tray was thermostated at 25 °C. A binary gradient of the eluents was used. Formic acid in water (0.1%) (A) and formic acid in methanol (0.1%) (B) were used; an isocratic elution was achieved for 2 min of 5% B, which was followed by a linear gradient of 98% B for 9 min, which was followed by an isocratic plateau for 1 min. The column was equilibrated back to 5% B in 0.5 min, and it was continued isocratically for 3.5 min.

The HESI source was operated in positive mode. The spray voltage was 4.5 kV, and the transfer capillary and probe heater temperatures were 320 and 400 °C, respectively. We used nitrogen gas as the source; we used 30 and 5 arbitrary units of sheath gas and auxiliary gas, respectively. The RF level of the S-lenses was 50; the automatic gain control target was set to 10^6^, and the resolution at *m*/*z* 200 was 35,000. Data were acquired in full scan mode (*m*/*z* 125–750).

### 4.4. HPLC Measurement to Determine the Amount of Cysteine and Glutathione

The jejunum was removed at the end of the experiment. Approximately 20 mg of the samples were put into an Eppendorf tube containing 500 µL of precipitating solution (100 mL containing 1.67 g of metaphosphoric acid (HPO_3_), 0.2 g of disodium EDTA, and 30 g of NaCl). The sample was homogenized through a Dounce tissue grinder and sonicated at 50 watts for 10 s (Huanghua Faithful Instrument Co., Cangzhou, China; LTD FSF-031S). The sample was kept in ice for 10 min and centrifugated at 12,000× *g* for 10 min. Fifteen µL of 0.3 M Na_2_HPO_4_ were added to 60 µL of the sample solution, and immediately after, 45 µL DTNB (20 mg of DTNB in 100 mL of 1% *w*/*v* sodium citrate solution) was added. The mixture was vortexed for 1 min, and after 5 min, the cysteine and glutathione were determined by RP-HPLC [63].

Analysis of the perfusates was performed using Agilent 1100 HPLC system that was equipped with a quaternary HPLC pump (G1311A), degasser (G1322A), an autosampler (G1313A), a thermostated column compartment (G1316A), and a UV-Vis detector (G1314A). Data were recorded and evaluated using Agilent ChemStation software (Rev.B.03.02-SR2). Reversed-phase Teknokroma NUCLEOSIL 100 C18 (4.6 mm × 250 mm, 5 µm particle size; (Teknokroma Analítica S.A., Sant Cugat del Vallès, Spain) was used for analytical separation.

**Figure A8:**
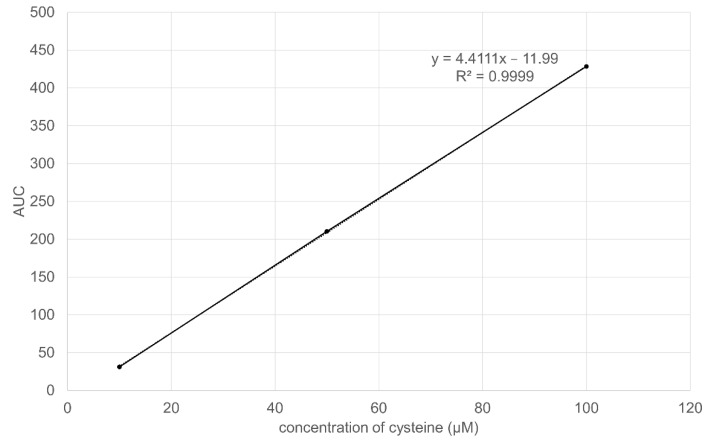
Calibration curve to determine the amount of cysteine in rat small intestine.

**Figure A9:**
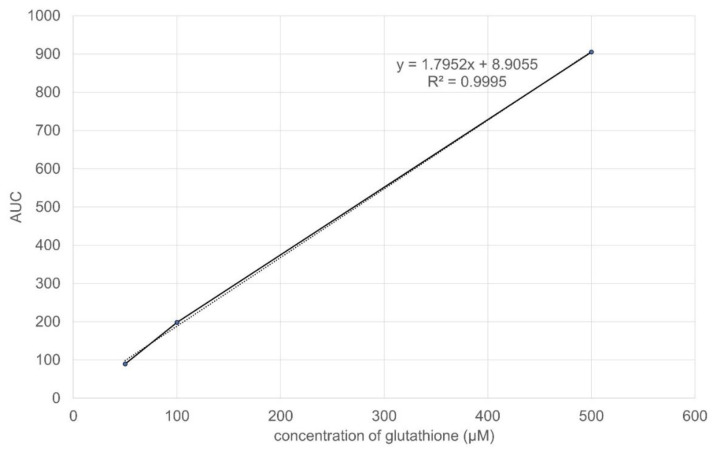
Calibration curve to determine the amount of glutathione in rat small intestine.

The mobile phase consisted of KH_2_PO_4_ solution (10 mM, pH = 6, adjusted with KOH) (buffer A) and buffer A containing acetonitrile (60% *v*/*v*) (buffer B). The gradient elution conditions were as follows: 100% buffer A for 10 min, which was followed by an increase to 100% buffer B in 15 min; this condition was maintained for 5 min. The gradient was returned to 100% buffer A in 3 min, and the column was regenerated with 100% buffer A for another 4 min before the injection of the next sample. The flow rate of the eluent was 1 mL/min. The volume of the samples was 20 µL, and the measurements were performed at 25 °C. Detection was performed at a wavelength of 330 nm [63]. The amount of cysteine and glutathione were calculated from standards (10–100 µM cysteine and 50–500 µM glutathione) that were prepared in the homogenization medium. We produced calibration equations also (Figure A8 and Figure A9).

### 4.5. Spectrophotometric Method to Determine CYP 3A4 Activity in the Small Intestine

To prepare the microsomes, the jejunum was removed from rats. It was minced and homogenized in an appropriate amount of 0.25 M sucrose containing 10 mM Tris hydrochloride (HCl) buffer at pH 7.4, then centrifuged at 600× *g* for 5 min, which was followed by 12,000× *g* for 10 min. The postmitochondrial supernatant was separated and mixed with solid CaCl_2_ so that its concentration in the given volume of supernatant was 8.0 mM, and then, it was centrifuged at 20,000× *g* for 20 min. The pellets were resuspended in a mixture of 150 mM KCl, 10 mM Tris hydrochloride (HCl) buffer, and they were centrifuged at 20,000× *g* for 20 min to obtain pinkish microsomal pellets, which were suspended in 0.5 mL of 0.1 M potassium phosphate buffer containing 20% (*v*/*v*) glycerol and store at −80 °C until their usage [63]. Quantification of proteins was performed by the Biuret assay.

The mixture of microsomal suspension (0.1 mL), erythromycin estolate (0.1 mL, 10 mM), potassium phosphate (0.6 mL, 100 mM, pH 7.4), and NADPH (0.1 mL, 10 mM) was incubated at 37 °C. It was centrifuged (2000× *g*; 10 min) to remove proteins. To 1.0 mL of this supernatant 1.0 mL of Nash reagent (2 M ammonium acetate, 0.05 M glacial acetic acid, and 0.02 M acetylacetone) was added, and it was heated in a water bath at 50 °C for 30 min. After cooling, the absorbance was measured at 412 nm. The activity was calculated from standards (100–1000 µM formaldehyde) that were prepared by substituting the sample with a standard solution in parallel with the other one. The CYP3A4 activity was expressed as µmol of formaldehyde that were obtained per milligram of protein per minute. The assay was also carried out with an inhibiting agent (10 µM ketoconazole) [64].

### 4.6. HPLC Measurement to Determine CYP2E1 Activity in the Small Intestine

The enzyme activity measurement of the CYP2E1 enzymes was performed from a small intestinal microsome suspension containing 1 mg/mL protein. The incubation mixture was prepared by mixing 0.4 mL homogenate, 0.4 mL 100 µM 4-nitrophenol (PNP), and 0.1 mL 10 mM NADPH. The incubation was carried out at 37 °C and for 30 min. The reaction was terminated by adding acetone and 1 M hydrochloric acid. An amount of the sample (0.1 mL) was mixed with 10 µL 1000 µM salicylamide (intestinal standard), and after vortex-mixing it, the sample was acidified by hydrochloric acid and extracted by diethyl ether. After drying and redissolving them in the eluent, the prepared samples were analyzed by an isocratic HPLC UV-Vis method. The measurement was at 250 nm with a flow rate of 1 mL/min. The eluent contained 22:77:1 *v*/*v*% acetonitrile: distilled water: glacial acetic acid. To this mixture, 3.036 g/L triethylamine was added, and the pH was set to 3. The quantity of the formed 4-nitrocatechol was measured [65].

### 4.7. Spectrophotometric Method to Determine COX Activity in the Small Intestine

The protocol for the preparation of microsomes was adapted from Zaveri et al. [64]. Quantification of proteins were performed by the Biuret assay. Microsomes (0.1 mg) were incubated with 2 mL Tris-HCl buffer (0.1 M, pH 8.6 adjusted with KOH) and N′N′N′*N*′-tetramethyl-p-phenylenediamine (40 µg/mL) at room temperature. The absorbance was recorded at 611 nm to establish the background rate of TMPD oxidation. Arachidonic acid (50 µM) in 5 µL ethanol was then added, and the absorbance changes were recorded for 30 s. The COX enzyme activity was expressed as µmol of TMBD that were obtained per milligram of protein per 30 s [66].

## 5. Conclusions

Similarly to 4-nitrophenol, paracetamol is also extensively metabolized in the small intestinal wall, thereby producing mainly conjugative derivatives (for the 4-nitrophenol, mainly glucuronide is formed, while at the paracetamol, glucuronide, and sulfate derivatives can be detected also). An important difference between the two compounds is the appearance of the oxidative metabolites in larger quantities for the paracetamol experiment. As degradation occurs, the cysteine and the mercapturate derivatives are also observable. The appearance of the cysteine and the measurement of the cysteine level from the intestinal homogenate might give us further information on the interplay of the cellular regulation function of cysteine and the paracetamol administration.

## Figures and Tables

**Figure A1:**
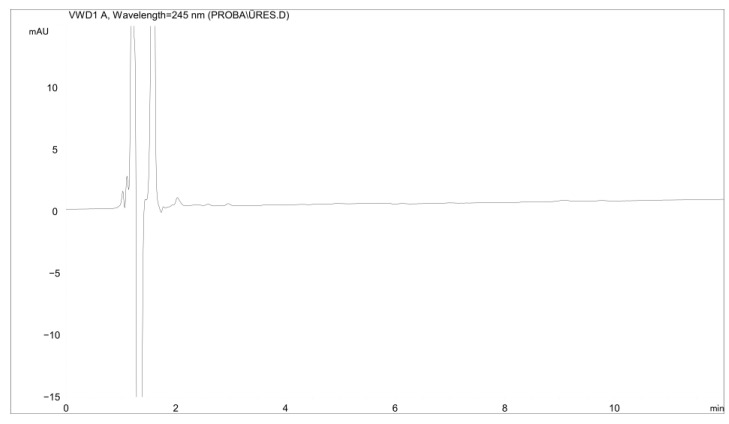
HPLC chromatogram of the blank small intestinal perfusate.

**Figure A10:**
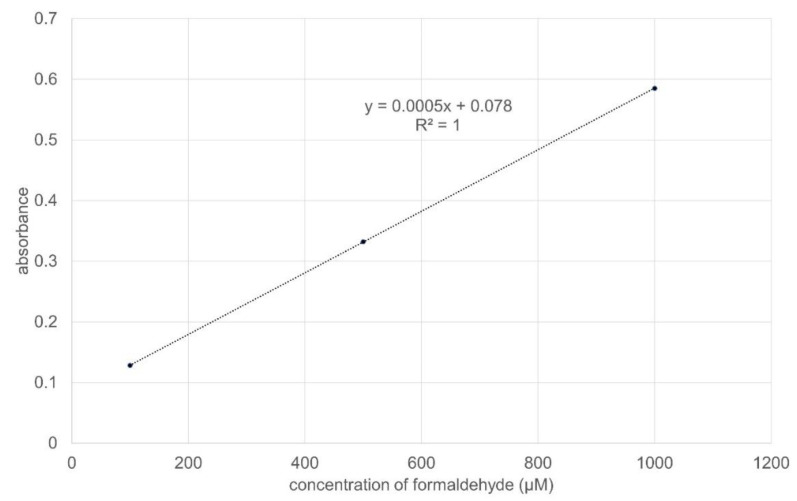
Calibration curve to determine the activity of CYP 3A4 enzyme in rat small intestine.

**Figure A11:**
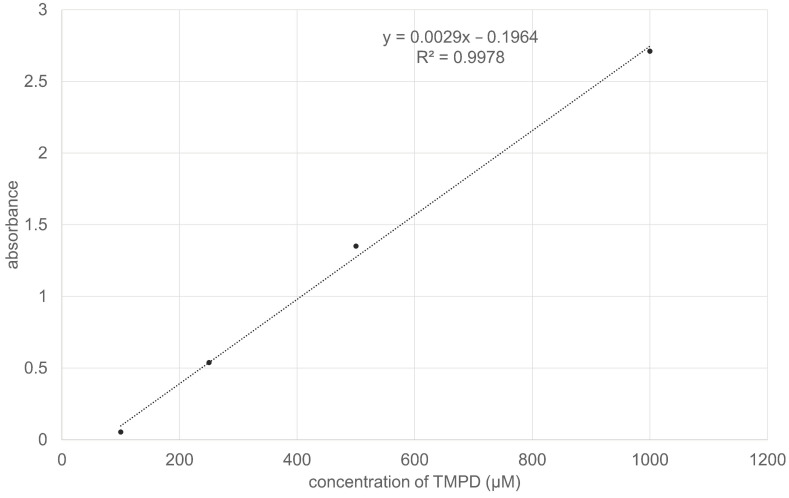
Calibration curve to determine the activity of COX enzyme in rat small intestine.

## Data Availability

All the data supporting reported results can be found in this manuscript.

## Appendix A

**Table A1:** Within-day and day-to-day precision of retention time and peak areas of paracetamol standard solutions in drug-free intestinal perfusate.

Concentration of Standard Solution (µM) Paracetamol	Intra-Day Precision of Retention Time (RSD %) (*n* = 5)	Intra-Day Precision of Peak Areas (RSD %) (*n* = 5)	Day-to-Day Precision of Retention Time (RSD %) (*n* = 5)	Day-to-Day Precision of Peak Areas (RSD %) (*n* = 5)
10	0.79	1.47	1.06	1.63
50	0.09	0.22	1.55	4.24
100	0.48	0.05	1.65	2.39
500	0.15	0.14	1.57	3.64
1000	0.25	0.1	1.28	7.30

Note: The precision of the peak areas were calculated from the ratios of the peak areas of paracetamol and theophylline. The values represent the mean of 5 independent measurements.

**Table A2:** Within-day and day-to-day precision of retention time and peak areas of paracetamol β-D-glucuronide standard solutions in drug-free intestinal perfusate.

Concentration of Standard Solution (µM) **Paracetamol β-D-glucuronide**	Intra-Day Precision of Retention Time (RSD %) (*n* = 5)	Intra-Day Precision of Peak Areas (RSD %) (*n* = 5)	Day-to-Day Precision of Retention Time (RSD %) (*n* = 5)	Day-to-Day Precision of Peak Areas (RSD %) (*n* = 5)
2	0.64	1.69	0.81	1.20
5	0.12	0.78	1.11	4.83
10	0.36	0.14	1.21	2.73
50	0.16	0.23	1.03	3.98
100	0.18	0.15	0.87	8.06

Note: The precision of the peak areas were calculated from the ratios of the peak areas of paracetamol β-D-glucuronide and theophylline. The values represent the mean of 5 independent measurements.

**Table A3:** Within-day and day-to-day precision of retention time and peak areas of paracetamol sulphate standard solutions in drug-free intestinal perfusate.

Concentration of Standard Solution (µM) Paracetamol Sulphate	Intra-Day Precision of Retention Time (RSD %) (*n* = 5)	Intra-Day Precision of Peak Areas (RSD %) (*n* = 5)	Day-to-Day Precision of Retention Time (RSD %) (*n* = 5)	Day-to-Day Precision of Peak Areas (RSD %) (*n* = 5)
2	0.80	2.62	1.29	2.31
4	0.19	1.38	2.03	4.05
6	0.60	0.75	2.13	2.80
8	0.15	0.60	1.70	3.90
10	0.30	0.22	1.60	8.35

Note: The precision of the peak areas were calculated from the ratios of the peak areas of paracetamol sulphate and theophylline. The values represent the mean of 5 independent measurements.

**Table A4:** Within-day and day-to-day precision of retention time and peak areas of paracetamol cysteine standard solutions in drug-free intestinal perfusate.

Concentration of Standard Solution (µM) Paracetamol Cysteine	Intra-Day Precision of Retention Time (RSD %) (*n* = 5)	Intra-Day Precision of Peak Areas (RSD %) (*n* = 5)	Day-to-Day Precision of Retention Time (RSD %) (*n* = 5)	Day-to-Day Precision of Peak Areas (RSD %) (*n* = 5)
2	0.92	1.81	1.27	1.69
4	0.11	0.39	1.77	4.65
6	0.59	0.64	1.92	3.01
8	0.16	1.28	1.64	4.40
10	0.29	0.66	1.47	8.12

Note: The precision of the peak areas were calculated from the ratios of the peak areas of paracetamol cysteine and theophylline. The values represent the mean of 5 independent measurements.

**Table A5:** Within-day and day-to-day precision of retention time and peak areas of paracetamol mercapturate standard solutions in drug-free intestinal perfusate.

Concentration of Standard Solution (µM) Paracetamol Mercapturate	Intra-Day Precision of Retention Time (RSD %) (*n* = 5)	Intra-Day Precision of Peak Areas (RSD %) (*n* = 5)	Day-to-Day Precision of Retention Time (RSD %) (*n* = 5)	Day-to-Day Precision of Peak Areas (RSD %) (*n* = 5)
2	0.89	0.75	1.80	3.13
4	0.11	1.21	2.54	5.62
6	0.69	0.17	2.72	2.81
8	0.25	0.13	1.97	4.10
10	0.37	0.11	1.73	8.30

Note: The precision of the peak areas were calculated from the ratios of the peak areas of paracetamol mercapturate and theophylline. The values represent the mean of 5 independent measurements.
